# Flourishing Scale: Adaptation and Evidence of Validity in a Chilean High School Context

**DOI:** 10.3389/fpsyg.2022.795452

**Published:** 2022-03-31

**Authors:** Marcos Carmona-Halty, Mauricio Marín-Gutierrez, Patricio Mena-Chamorro, Geraldy Sepulveda-Páez, Rodrigo Ferrer-Urbina

**Affiliations:** Escuela de Psicología y Filosofía, Universidad de Tarapacá, Arica, Chile

**Keywords:** flourishing, psychometric analyses, high school students, Chilean students, gender invariance

## Abstract

This study aimed to adapt the Flourishing Scale to a Chilean high school context and provide evidence of its validity. Data were collected from 1,348 students (52% girls) from three different Chilean schools. The results of confirmatory factor analysis (CFA) supported a one–factor solution, multiple–group CFA supported gender invariance, and structural equation model indicated that the FS is related to positive and negative academic feelings. Overall, the evidence indicates that the Flourishing Scale adapted to the high school context is an instrument that produces valid and reliable scores in our high school Chilean sample.

## Introduction

Positive education is an emerging area of study aimed at encouraging—without ignoring the negative aspects inherent in all human activity—members of the educational community to flourish and develop their full potential (Jacobs and Renandya, 2019). More specifically, positive education is a discipline that emerges from positive psychology and aims to complement the traditional emphasis on developing academic skills with initiatives to promote well–being and optimal functioning (Seligman et al., 2009). In this line, recent research has shown that the development of personal strengths and resources are potential variables for increasing performance and other desirable outcomes in the high school setting (e.g., Steinmayr et al., 2018; Widlund et al., 2018; Su et al., 2019). In addition, the efficacy of programs aimed at increasing levels of well–being and reducing depressive symptomatology, which favor academic performance, has been confirmed (e.g., Shoshani and Steinmetz, 2013; Shoshani and Slone, 2017; Schoeps et al., 2018).

One of the concepts that has received increasing attention from educational contexts is the so–called *flourishing* (e.g., De la Fuente et al., 2017; Shoshani and Slone, 2017; Datu, 2018; Garzón–Umerenkova et al., 2018; Datu et al., 2019; Chamizo–Nieto et al., 2021; Holliman et al., 2021). Flourishing is synonymous with a high mental well–being level and reflects positive mental health and positive development (Huppert and So, 2013; Hone et al., 2014). More specifically, flourishing is the combination (in a single construct) of feeling good and functioning effectively in one’s life. The first refers to feel interest in and a commitment to the activities of daily living, self–confidence, and affect, while the second refers to feeling in control of the course of one’s life, having a purpose, and establishing and maintaining positive relationships with others (Ryff and Singer, 1998; Keyes, 2002; Huppert, 2009; Huppert and So, 2013; De la Fuente et al., 2017).

Recent positive education research has shown that flourishing is positively related to desired academic outcomes, such as performance (Datu, 2018), personal resources (Ouweneel et al., 2011), engagement (Datu, 2018), achievement goal orientation (Datu et al., 2019), adaptability and social support (Holliman et al., 2021), positive teacher–student relationships (Chamizo–Nieto et al., 2021), basic psychological needs (Herrera et al., 2021), and passion for learning (Chen et al., 2021). Conversely, it is negatively related to undesired academic outcomes, such as depression and distress (De la Fuente et al., 2017), procrastination (Garzón–Umerenkova et al., 2018), and psychotic experiences (Oh et al., 2021). Together, these studies show that flourishing is a key construct applicable to the high school context and to the aims of positive education. Therefore, flourishing could help understand the processes underlying the optimal functioning of children and adolescents in school contexts.

One of the most widely used instruments to evaluate flourishing is the Flourishing Scale (FS) developed by Diener et al. (2010). This scale is a brief self–reported measurement that assesses the key components of psychosocial well–being: meaning and purpose in life, supportive and rewarding relationships, engaged and interested, contribute to the well–being of others, competency, self–acceptance, optimism, and being respected (Diener et al., 2009). Initial validation studies support FS as a one–factor solution with adequate psychometric properties (see Diener et al., 2010). More recently, additional validation studies have supported its psychometric properties (e.g., Romano et al., 2020; Martín–Carbonell et al., 2021; Tan et al., 2021) and shown its cross–cultural validity (e.g., Brazil—da Fonseca et al., 2015; China—Lin, 2015; France—Villieux et al., 2016; Egypt—Salama–Younes, 2017; India—Singh et al., 2016; New Zealand—Hone et al., 2014; Russia—Didino et al., 2019).

Despite the contribution that the studies have made to flourishing research, more research efforts are needed, specially, in Spanish–speaking South American countries where minimal research was done to assess the psychometric properties of FS (e.g., Colombia—Martín–Carbonell et al., 2021; Peru—Cassaretto and Martínez Uribe, 2017). The present study attempts to fill the gap on the scarcity of flourishing measures by adapting the FS to the Chilean high school context and examining its psychometric properties. We hope to contribute to increasing the scarce research on positive education in South American countries. More specifically, we aim to adapt the FS to the usual conditions of Chilean high school students and provide evidence of its validity following both a within–network and between–network construct validity. The first refers to assessing reliability, factor structure, and gender invariance, while the second refers to assessing the extent to which flourishing is associated with theoretically related constructs. In this line, given that the FS measures (only) the psychosocial components of well–being, the Scale of Positive and Negative Experiences (SPANE) —developed by Diener et al. (2010)— complements this indicator by measuring a range of positive and negative emotions and feelings in a specific time range (for example, during the past 4 weeks). Accordingly, the FS score has shown positive and negative significant relationships with the positive and negative feelings dimensions of the SPANE, respectively. For example, 0.69 and –0.48 (Giuntoli et al., 2017); 0.67 and –0.47 (Howell and Buro, 2015); and 0.58 and –0.42 (Silva and Caetano, 2013).

Based on the arguments presented, we hypothesize the following: The FS adapted to the high school context will demonstrate adequate psychometric properties in a sample of Chilean high school students. Also, we expect positive and negative relationships between FS scores and study–related positive and negative feelings (measured with the SPANE), respectively.

## Method

### Sample

The sample comprised 1,348 (52% girls) Chilean high school students between grades 7–12 (i.e., 13–18 years old, *M* = 15.04, SD = 1.43). The students were from three different secondary schools (each of them hosted approximately 600 students) from two urban centers in the country’s northern regions: Arica and Iquique. Of 1,348 students, 17% were 13 years old, 19% were 14 years old, 18% were 15 years old, 21% were 16 years old, 22% were 17 years old, and 3% were 18 years old. In addition, 13% correspond to low, 79% to medium, and 8% to high socioeconomic levels.

### Instruments

The *Flourishing Scale* (Diener et al., 2010) is composed of eight--item. Each item is rated by respondents using a 7--point Likert scale (1 = *strongly disagree*, 7 = *strongly agree*). In this study, 3 expert judges were asked to compare both the Spanish and English language version of the FS (available on Ed Diener’s website^1^) to establish whether both versions did not differ from each other. Furthermore, they checked the instrument’s legibility. Subsequently, the FS was adapted to the educational setting of the students following the recommendations described in the literature associated with the adaptation of instruments (see Muñiz et al., 2013; Vallejo–Medina et al., 2017). More specifically, a rewording of the items from the general context to the school context was conducted. For example, “*I am engaged and interested in my daily activities*” was changed to “*I am engaged and interested in my daily school activities*.” Finally, a pilot test was conducted with the FS adapted version (see Table 1) where 30 Chilean high school students were encouraged to answer the scale and indicate possible comprehension issues. At this stage, none of the participants expressed problems with understanding the items or the answering format of the FS.

**TABLE 1:** Descriptive and reliability statistics information of the flourishing scale.

	Descriptive statistics				Reliability statistics			CFA factor loadings	S.E.
	Mean (SD)	S	K	W	CHI	α if item is dropped	ω if item is dropped		
1. I lead a purposeful and meaningful school life.	5.83 (1.359)	−1.187	0.977	0.811*	0.631	0.850	0.852	0.744*	0.015
2. My social relationships at school are supportive and rewarding.	5.73 (1.409)	−1.182	1.027	0.824*	0.499	0.865	0.865	0.598*	0.020
3. I am engaged and interested in my daily school activities.	5.88 (1.250)	−1.140	0.986	0.819*	0.679	0.845	0.846	0.784*	0.014
4. At school I actively contribute to the happiness and well-being of others.	5.81 (1.237)	−1.043	0.861	0.840*	0.592	0.854	0.856	0.692*	0.017
5. I am competent and capable in school activities that are important to me.	6.30 (0.972)	−1.667	3.151	0.722*	0.607	0.853	0.854	0.748*	0.017
6. At school I am a good person and live a good life.	6.10 (1.124)	−1.512	2.592	0.770*	0.663	0.847	0.848	0.769*	0.014
7. I am optimistic about my school future.	6.07 (1.292)	−1.771	3.211	0.729*	0.665	0.846	0.847	0.771*	0.015
8. People at school respect me.	5.92 (1.238)	−1.351	1.848	0.805*	0.613	0.853	0.854	0.712*	0.016
Flourishing	47.68 (7.110)	−19.04	16.47	0.901*					

*p < 0.001; SD, standard deviation; S, skewness standardized; K, kurtosis standardized; W, Shapiro–Wilk test; CHI, corrected homogeneity index; CFA, confirmatory factor analysis; and S.E., standard error.

The *Scale of Positive and Negative Experiences* (Diener et al., 2010) is composed of 12 items. Each item is rated by respondents using a 5–point Likert scale (1 = *never*, 5 = *always*). The scale is integrated by two subscales (six items each): positive (e.g., “*I have had pleasant feelings*”) and negative (e.g., “*I have had unpleasant feelings*”) feelings. This study used an adaptation to the Chilean high school context of the original SPANE, which demonstrated adequate psychometric properties (see Carmona–Halty and Villegas–Robertson, 2018). In our sample, internal consistency —for alpha and omega index— was 0.931 and 0.931, for study–related positive feelings, and for study–related negative feelings was 0.849 and 0.855, respectively.

### Procedure

The procedure included contacting the principals of schools to explain to them the research’s aim, scope, and needs. Once the proposal was accepted, a written authorization was requested from the principals, students, and parents. Data collection was carried out in group sessions of 25 students through an electronic procedure. For this purpose, each student had a computer at their disposal where the questionnaires had been previously uploaded. The students took about 10 min to answer the questionnaire and data collection lasted approximately 3 weeks.

### Analysis

Sequential analyses were conducted using Jamovi 1.2 (The Jamovi Project, 2020) and Mplus 8.2 (Muthén and Muthén, 1998/2017). First, mean scores, standard deviation, standardized skewness and standardized kurtosis, gender differences, and Shapiro–Wilk test were calculated. Second, the internal consistency was estimated using Cronbach’s alpha (α) and McDonald’s omega (ω) coefficients, the corrected homogeneity index, and the alpha and omega indexes if any of the items were eliminated. Third, to determine whether the model proposed by the FS adequately represents the data collected, a confirmatory factor analysis (CFA) was performed using the weighted least square with mean estimation method (WLSMV) —which is robust to significant deviations from the normal distribution— and the polychoric correlation matrix. The model fit was interpreted according to the cut–off points proposed by Schreiber (2017) (e.g., CFI > 0.95; TLI > 0.95; RMSEA < 0.06). Fourth, to explore gender invariance, a multiple–group CFA was performed, where three levels of equivalence (i.e., configural invariance, metric invariance, and scalar invariance) were evaluated (Chen and West, 2008), using changes in CFI and RMSEA (Δ < 0.010) as criteria to determine whether measurement invariance was established (Cheung and Rensvold, 2002; Chen, 2007; Dimitrov, 2010). Finally, to examine the criterion validity of the FS, a structural equation model (SEM) was conducted between the covariations of positive and negative feelings and the FS score.

## Results

### Descriptive Analysis

Table 1 shows the descriptive statistics for the FS at item level, including reliability and factor loading as they emerged in the CFA analysis described below. The Shapiro–Wilk test showed that none of the items had a normal distribution. Following previous research (e.g., Diener et al., 2010), gender differences were considered. However, independent sample *t*-test reveal that there are not statistical significance differences between boys’ (*M* = 6.015, SD = 0.728) and girls’ (*M* = 5.929, SD = 0.988) FS scores: *t*_(1346)_ = 1.947, *p* > 0.05.

### Within–Network Construct Validation

**FIGURE 1:**
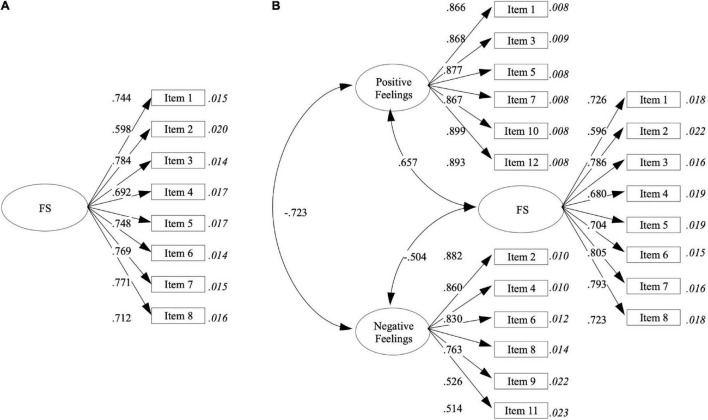
Graphical representation of flourishing scale model **(A)** and the SEM covariate between FS and SPANE **(B)**. Values in italics correspond to the standard error.

The FS adapted to the Chilean high school context showed adequate internal consistency for Cronbach’s alpha (α = 0.868) and McDonald’s omega (ω = 0.869) index. In addition, as shown in Table 1, the results of the corrected homogeneity index suggests that it is not necessary to delete any items. Table 2 (M1) and Figure 1A shows the CFA results for a model assuming one latent factor underlying all FS items. According to the standards recommended by Schreiber (2017), this model showed adequate fit index, reflecting a sufficient explanation for the observed covariate matrix. Indeed, the factorial loadings show adequate representations (λ > 0.50). In addition, the multiple–group CFA shows that the differences in the CFI and RMSEA —across the three invariance models (i.e., configural, metric, and scalar)— were lower than 0.010, which indicates gender invariance.

**TABLE 2:** Fit Indexes for single–group and multiple–group CFA of the flourishing scale.

	χ^2^	*df*	χ^2^/*df*	RMSEA	90% CI	CFI	TLI	SRMR	CMs	Δ CFI	Δ RMSEA
**Single–group CFA**											
M1 One factor solution	215.24	20	107.62	0.085	[0.075, 0.096]	0.976	0.967	0.024	–	–	–
**Multiple–group CFA**											
M2 Configural invariance	261.60	40	5.415	0.091	[0.080, 0.101]	0.946	0.924	0.037	–	–	–
M3 Metric invariance	278.20	47	5.919	0.085	[0.076, 0.095]	0.944	0.933	0.055	M2–M3	0.002	0.006
M4 Scalar invariance	313.47	54	5.805	0.084	[0.076, 0.094]	0.937	0.934	0.065	M3–M4	0.007	0.001

*χ2, Chi-square; df, degree of freedom; RMSEA, root mean square error of approximation; CI, 90% confidence interval; CFI, comparative fit index; TLI, Tucker–Lewis index; SRMR, standardized root mean square residual; and CMs, comparisons between models.*

### Between–Network Construct Validation

The SEM model showed satisfactory comparative and absolute fit indexes: χ^2^ (167, 1,348) = 959.437, *p* < 0.05; CFI = 0.980; TLI = 0.978; RMSEA = 0.059, 90% CI (0.056–0.063). As shown in Figure 1B, there are positive (γ = 0.657, *p* < 0.001) and negative (γ = −0.504, *p* < 0.001) relationships between FS scores and positive and negative feelings, respectively.

## Discussion

The current study aimed to adapt the FS to the Chilean school context and obtain evidence of its validity to address the lack of measures and facilitate flourishing research in educational settings.

Our results are consistent with previous research in terms of the reliability indices and factor structure of the FS (e.g., Diener et al., 2010; Silva and Caetano, 2013; Howell and Buro, 2015; Villieux et al., 2016; Giuntoli et al., 2017; Checa et al., 2018). Also, gender invariance was demonstrated, leading to the conclusion that flourishing can be measured with the same precision in boys and girls, which is consistent with recent studies (e.g., Romano et al., 2020; Martín–Carbonell et al., 2021; Tan et al., 2021). Furthermore, students who report higher levels of flourishing are more likely to experience study–related positive feelings (e.g., happiness, pleasure, and satisfaction) and less likely to experience study–related negative feelings (e.g., sadness, displeasure, and anger).

The main strength of the present study is the large sample used. However, there are also some limitations that highlighting possible paths for future research. First, we use a convenience sample, and our results should be generalized with caution. Therefore, future research could use a representative and diverse sample to generalize its results to the Chilean high school population. Second, the cross–sectional nature of the design does not allow to prove the temporal stability of the FS. Therefore, future research may include longitudinal designs to analyze their stability and temporal invariance. Third, the use of self–report data may increase the probability of incurring common method variance. Hence, it would be interesting to move toward an external measure of flourishing.

The results suggest that the FS adapted to the Chilean high school context can thus be considered a valid and reliable tool for researchers and practitioners. For researchers, this measure contains only eight items and is, therefore, a short and practical instrument, which offers a broad view of positive and healthy functioning that has been shown to be important for students’ optimal functioning. For practitioners, high schools can take advantage of this measure by including it within their diagnosis and monitoring activities. That is, knowing the state of their student’s flourishing will allow them to design and deploy properly grounded actions to foster their well–being and contribute to the building of a healthy and thriving school community.

## Data Availability Statement

The raw data supporting the conclusions of this article will be made available by the authors, without undue reservation.

## Ethics Statement

The studies involving human participants were reviewed and approved by the Comité Ético Científico of the Universidad de Tarapacá (CEC-UTA). Written informed consent to participate in this study was provided by the participants’ legal guardian/next of kin.

## Author Contributions

All authors contributed equally to the research design and wrote the manuscript.

## Conflict of Interest

The authors declare that the research was conducted in the absence of any commercial or financial relationships that could be construed as a potential conflict of interest.

## Publisher’s Note

All claims expressed in this article are solely those of the authors and do not necessarily represent those of their affiliated organizations, or those of the publisher, the editors and the reviewers. Any product that may be evaluated in this article, or claim that may be made by its manufacturer, is not guaranteed or endorsed by the publisher.

## Footnotes

1
https://eddiener.com
